# Plant Lipid Bodies Traffic on Actin to Plasmodesmata Motorized by Myosin XIs

**DOI:** 10.3390/ijms21041422

**Published:** 2020-02-20

**Authors:** Manikandan Veerabagu, Laju K Paul, Päivi LH Rinne, Christiaan van der Schoot

**Affiliations:** Department of Plant Sciences, Norwegian University of Life sciences, N-1432 Ås, Norway; manikandan.veerabagu@nmbu.no (M.V.); paul.laju@gmail.com (L.K.P.);

**Keywords:** F-actin, *Arabidopsis*, cytochalasin D, cytoskeleton, lipid body/droplet, 3KO myosin XI knockout, oleosin, plasmodesmata, *Populus*

## Abstract

Late 19th-century cytologists observed tiny oil drops in shoot parenchyma and seeds, but it was discovered only in 1972 that they were bound by a half unit-membrane. Later, it was found that lipid bodies (LBs) arise from the endoplasmic reticulum. Seeds are known to be packed with static LBs, coated with the LB-specific protein OLEOSIN. As shown here, apices of *Populus* *tremula* x *P. tremuloides* also express *OLEOSIN* genes and produce potentially mobile LBs. In developing buds, *PtOLEOSIN (PtOLE)* genes were upregulated, especially *PtOLE6,* concomitant with LB accumulation. To investigate LB mobility and destinations, we transformed *Arabidopsis* with *PtOLE6-eGFP*. We found that PtOLE6-eGFP fusion protein co-localized with Nile Red-stained LBs in all cell types. Moreover, PtOLE6-eGFP-tagged LBs targeted plasmodesmata, identified by the callose marker aniline blue. Pharmacological experiments with brefeldin, cytochalasin D, and oryzalin showed that LB-trafficking requires F-actin, implying involvement of myosin motors. In a triple myosin-XI knockout (*xi-k/1/2*), transformed with *PtOLE6-eGFP*, trafficking of PtOLE6-eGFP-tagged LBs was severely impaired, confirming that they move on F-actin, motorized by myosin XIs. The data reveal that LBs and OLEOSINs both function in proliferating apices and buds, and that directional trafficking of LBs to plasmodesmata requires the actomyosin system.

## 1. Introduction

The presence of cytosolic lipid inclusions was first noticed in the late 19th century in parenchyma cells of roots and shoot-axils, and seeds of *Ricinus* [1]. It is now well-established that cytosolic “oil drops,” as described by the early plant cytologists, are of universal occurrence [2,3,4]. However, their origin, structure, and precise composition remained elusive for many decades, which is reflected in the plethora of descriptive names that sprang up, including lipid droplets, fat droplets, oil droplets, oil globules, oleosomes, spherosomes, oil bodies, and lipid bodies [2,5]. An important milestone was the discovery in the early 1970s that the single “dark-line” surrounding the “spherosomes” of peanut seeds, visible in electron micrographs, was not a nonspecific adsorption layer but a half unit-membrane [6]. It was suggested that spherosomes may result from the local deposition of oil between the leaflets of unit-membranes, a hypothesis that was supported by the presence of structural membrane proteins with extreme insolubility [6]. Later investigations corroborated these inferences and showed that the site of their formation is the endoplasmic reticulum (ER) [2,7,8,9].

Although the listed terms are still used interchangeably, the naming is not entirely trivial, and may reflect different research interests. For example, from the perspective of emulsion physics the term “lipid droplet” (LD) refers to an oil inclusion that is coated by phospholipid as a surfactant to minimize the surface tension at the oil-water interface, thereby preventing coalescence [4,10]. Similarly, in lipid storage physiology, LDs might refer to lipid stores that function in cellular lipid and energy homeostasis, and detoxification [11,12]. In some mammalian cell types micron-sized lipid depositions, referred to as chylomicrons, can form within the ER lumen [13]. Although the term LD is most widely adopted, especially in non-plant research, in a cell-biological context the term “lipid body” (LB) has clear merits. It discriminates plant LBs from the lipid inclusions in animal cells, which for example, also include simple dietary chylomicrons [14]. Moreover, the term LB emphasizes the structural and functional aspects of the LB surface that define organellar interactions [5].

Regardless of the naming, all genuine LBs share the unique topology of an ER-derived half unit-membrane, embedded with structural proteins, that surrounds a core of neutral lipids [2]. The structural “resident” proteins provide stability and allow docking of lipases and other enzymes, while various cytosolic proteins associate with LBs, for example, through amphipathic domains, lipid anchors and N-terminal hydrophobic domains [15,16,17,18]. The consensus is that LBs form at specialized sites of the ER by the deposition of triacyl glycerides (TAGs), a process referred to as “nucleation” [10]. In yeast, mammalian cells, and plant seeds, LB formation is promoted by seipins, membrane spanning proteins that stabilize the LB-ER junction [19,20] and facilitate TAG transport into the budding LB [21,22]. This requires the local presence of diacylglycerol acyltransferase 1 (DGAT1) or DGAT2, enzymes that catalyze TAG biosynthesis [23,24,25]. At a critical concentration, TAGs and bilayer phospholipids unmix, resulting in an oil lens that gives rise to a budding LB [9,26].

In higher eukaryotes, LBs are pinched off into the cytosol, giving rise to independent and potentially mobile organelles, while some may remain connected or become reconnected to the ER via lipidic bridges or molecular tethers [27]. By implication, this part of the LB population is not available for translocation. In yeast, none of the LBs is mobile as they remain attached via lipidic bridges to the ER [8,21,28]. Lipidic bridges not only allow TAG import, but also the migration of integral membrane proteins, including the TAG biosynthesis enzymes glycerol-3-phosphate acyltransferase 4 (GPAT4) and DGAT2 [8,28,29]. Both enzymes are predicted to contain transmembrane domains (TMDs) [23,24,30,31], which would make embedment in a monolayer unlikely. However, it has been proposed that the two predicted TMDs of DGAT2 form a single domain within the ER bilayer [4,17,24]. In case of GPAT4, the predicted multiple TMDs might form a hydrophobic loop that becomes embedded under strain in the ER [28]. By moving TAG biosynthesis enzymes onto LBs, biosynthesis could continue even after LBs are pinched off, while at the same time making them available for transport.

Mobile LBs may also form as depicted in the “bicelle or hatching model.” Here, the complete oil lens is cut out, resulting in a LB monolayer composed of both ER leaflets [32,33]. Alternatively, in the “vesicular budding model“, tiny vesicles are formed that are tethered to the ER, possibly involving coatomers [34,35], while a shuttle mechanism transfers TAGs into its bilayer. As a result, the expanding LB contains an aqueous inclusion that is surrounded by the inner ER leaflet. Inevitably, the specific mechanism that gives rise to LBs will influence the kinds of phospholipids and embedded proteins that are present, which might determine if transport mechanisms can latch on to them. Whether in plants LBs are produced by the above mechanisms is not clear.

Plant LBs can contain a variety of proteins, including OLEOSINS, CALEOSINS, STEROLEOSINS [2,5], LDAPs, and LDIP [36]. In seeds, the extraction of LBs from the ER is controlled solely by the LB-specific integral protein OLEOSIN [3,7,37]. OLEOSINs are small 15–26 kD proteins that are inserted co-translationally into the ER-membrane, with the termini located in the cytosol, and the central hydrophobic hairpin positioned under strain in the ER bilayer [37] similarly to GPAT4 [28]. From this unstable position the OLEOSIN molecule will diffuse into an emerging oil lens, extracting it from the ER while relaxing its hydrophobic hairpin in the lipid core. The length of the hairpin is instrumental, as shorter hairpins reduce their LB targeting [37]. Although the N- and C-termini are not essential for targeting, they are important for providing full coverage of the LB surface, thereby preventing coalescence with adjacent LBs via steric hindrance and electronegative repulsion. OLEOSINs are believed to be desiccation-related LB proteins that, with some exceptions, are seed-specific [38]. Seeds are closely packed with mostly static LBs that function to store energy and lipid precursors for membrane biogenesis [38,39]. Their proteome is probably restricted, and only enriched during germination with enzymes that mobilize the lipids [40].

The strong focus on seed LBs has led to a relative neglect of their presence and potential roles in post-embryonic tissues. The solitary LBs in these tissues interact with other organelles, for example, mitochondria, which are often captured in microscopic images. Such interactions might explain why the proteomes of isolated LBs tend to be enriched in signaling molecules that are characteristic of these organelles. In mammalian cells this is the default situation, and frequent “kiss-and-run” encounters between LBs and other organelles [41] result in rich and variable LB cargos [42,43,44,45]. For example, these encounters might result in the exchange of lipophilic signals, monolayer-embedded proteins, and proteins that are electrostatically bound. Moreover, opportunistic hitchhikers might piggyback on trafficking LBs, for example, by attaching to them electrostatically [5,46].

Among the post-embryonic tissues, shoot apices and buds of perennial plants are of considerable interest, as LBs play an intriguing role in the seasonal phenology. In autumn, the shoot apex encloses itself in a terminal bud and resumes growth in spring. While the growing apex may contain few LBs, developing buds accumulate substantial amounts of them [47,48]. Although this is mostly overlooked in the LB literature, there are interesting parallels with orthodox seeds. Like seeds, buds desiccate, establish dormancy, and express genes that confer tolerance to dehydration and frost [49,50]. Similarly, bud LBs store energy and provide lipid precursors for membrane biogenesis during regrowth. On the other hand, buds do not completely desiccate [51], are less densely packed, and LBs may retain some mobility that appears crucial in dormancy release [47,52]. Release from dormancy is mediated by LBs that move to plasmodesmata (PD) in the shoot apical meristem (SAM), which during dormancy establishment becomes physically closed by callose deposits in dormancy-sphincter-complexes (DSC) at PD orifices [5,48,53]. The LB-PD alignment seems crucial, as it results in removal of callose and functional restoration of PD conduits, probably because the LB cargo contains callose-hydrolyzing enzymes, γ-clade 1,3-β-glucanases. These enzymes are proactively produced in developing buds and stored at the accumulating LBs for later use. The functionality of this mechanism was further demonstrated by transient expression analyzes in tobacco leaves, which showed that eGFP-tagged 1,3-β-glucanases localized to the PD [52]. PD-targeting by LBs may not only play a role in dormancy release, but also in proliferating apices, because they are centers of morphogenesis and de novo pattern formation, which involve continuous symplasmic exchange of signals and metabolites [47,54,55]. However, it remains unknown how LBs traffic across the cytoplasm from random positions to PD and other putative destinations.

Directional movement of LBs and other organelles in non-plant systems is known to occur on microtubules. In Drosophila and mammalian cells, such movement is driven by the plus-end directed motor protein kinesin and the minus-end directed dynein [56,57,58,59]. In plants, directional transport of many organelles is driven by the actomyosin system. For example, transport of endomembrane vesicles, mitochondria, peroxisomes, vacuolar structures, RNA processing bodies, ER, Golgi stacks, and secretory vesicles is dependent on F-actin [60,61,62,63,64,65,66,67].

Here we investigated the presence of LBs in actively growing apices and buds of *Populus*, and the mechanism by which they might be trafficked through the cytosol to target PD. As the shoot meristem of this species is not amenable for live microscopical studies, we used *Arabidopsis* to study LB motility. To this purpose, we first identified *Populus* homologs of the *Arabidopsis OLEOSIN* genes and analyzed the extent to which these LB-marker genes were expressed in proliferating apices and developing buds. Apices expressed eight of the nine *PtOLEOSIN* genes and upregulated particularly *PtOLE6* gene during LB accumulation. Genetic transformation of *Arabidopsis* with the highly expressed *PtOLE6* yielded genuine PtOLE6-eGFP-labeled LBs that stained specifically with the neutral lipid stain Nile Red, and targeted PD-orifices. Pharmacological experiments showed that directional LB movement required F-actin. In a triple myosin-XI knockout (*xi-k/1/2*), transformed with *PtOLE6-eGFP*, LB trafficking was severely impaired, indicating that directional movement of LBs is on F-actin, motorized by myosin XIs.

## 2. Results

### 2.1. Shoot Developmental Status in Relation to LBs

**Figure 1:**
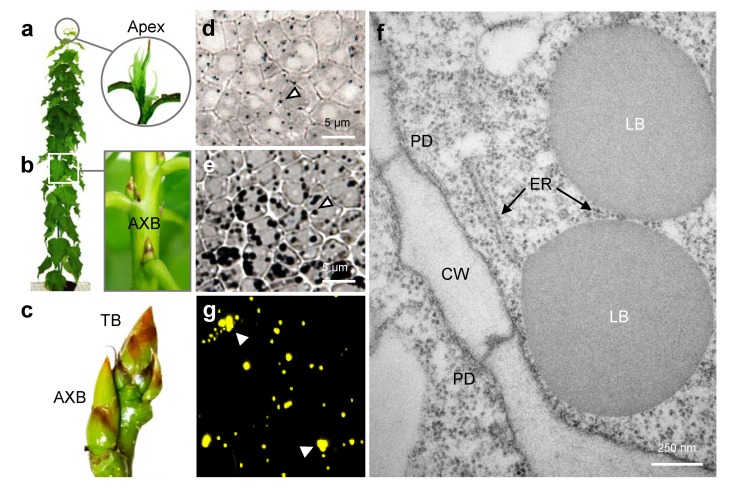
Lipid bodies (LB) are common constituents of shoot meristem cells in hybrid aspen. Under a long photoperiod (**a**,**b**,**d–f**), the actively growing shoot apex (**a**) (circled) harbors the shoot apical meristem, which possesses a few LBs (**d**) that are visible as dark blue spots after staining with the lipid stain Sudan Black (white arrowhead). (**b**) Quiescent axillary buds (AXB) (boxed) host numerous, relatively large LBs, which stain intensely black (**e**) (white arrowhead). In TEM micrographs, LBs could be seen (**f**) in the proximity of the plasma membrane and plasmodesmata (PD). Under a short photoperiod (**c,g**) the apex forms a terminal bud (TB), whereas young AXBs enlarge (**c**) and accumulate LBs. Isolated LBs from dormant buds (**g**) retained their spherical shape, although some coalescence was observed (white arrowheads), stained with the neutral lipid stain Nile Red. Endoplasmic reticulum (ER); cell wall (CW).

Hybrid aspen (*Populus tremula* × *P. tremuloides*) is a perennial woody species of the temperate climate zones. The shoot apex harbors the apical meristem (SAM), which is hidden from view by the youngest overarching leaves (Figure 1a). Typically, the shoot apex produces nascent axillary buds (AXBs) in the axils of emerging leaves, which gradually develop toward completion (Figure 1b). AXBs are mature at the so-called bud maturation point, when about 12 new internodes and leaves with their AXBs have arisen above it from the growing shoot tip [68]. At maturity, AXBs contain a pre-formed miniature shoot system that has ceased development and remains inactive until after winter. Due to this postponement of AXB outgrowth, the shoot system remains unbranched during the first growing season (Figure 1b). Under a short photoperiod, the SAM produces a terminal bud (TB; Figure 1c) that adopts the developmental program of AXBs [68] and establishes a dormant state [52,69]. Likewise, all the young developing AXBs (Figure 1c) are arrested in a dormant state [68].

We used light microscopy to visualize LBs in tissue sections that were stained with the neutral lipid stain Sudan Black. In the actively proliferating apex, SAM cells contained a limited amount of dark blue-stained LBs (Figure 1d). In contrast, in AXBs, the rib meristem that is subjacent to the SAM, numerous intensely black LBs were crowding the available cytoplasmic space around the nuclei (Figure 1e). Such LB accumulation was present in all buds, whether quiescent or dormant. In brief, although excessive amounts of LBs are produced in the developing buds, especially in the rib meristem, they are also continuously produced at low levels by the proliferating SAM.

Transmission electron microscopy of the SAM of quiescent AXBs confirmed that the Sudan Black stained deposits were LBs. They were spherical and evenly-sized (ca. 1.0 µm), with a smooth phospholipid monolayer surrounding a grey osmiophilic matrix, as exemplified in Figure 1f. The LBs were present as free cytosolic organelles with no sign of LB-LB coalescence. Occasionally, LBs appeared in proximity to ER strands. In the rib meristem the LBs were slightly enlarged (Figure 1d,e). Nile Red-stained LBs isolated from dormant buds mostly retained their spherical shape (Figure 1g).

### 2.2. Identification and Expression of Populus OLEOSIN Genes

Previous work showed that LBs play an important role in releasing dormancy. At the end of the growing season, SAM function is arrested by the installation of callose-containing DSCs at all PD. The DSCs uncouple all SAM cells from their shared symplasmic network, thereby locking the SAM in a dormant state [47,54]. Reversely, dormancy release involves displacement of LBs to the PD, disappearance of DSCs, and restoration of dye-coupling between the cells. This requires callose hydrolysis by LB-resident 1,3-β-glucanase [47,48,52]. Although this showed that LBs can target PD to deliver enzymes that modify the PD channel, it has remained unknown which LB proteins are involved in targeting, and how LBs move toward the PD.

**Figure 2:**
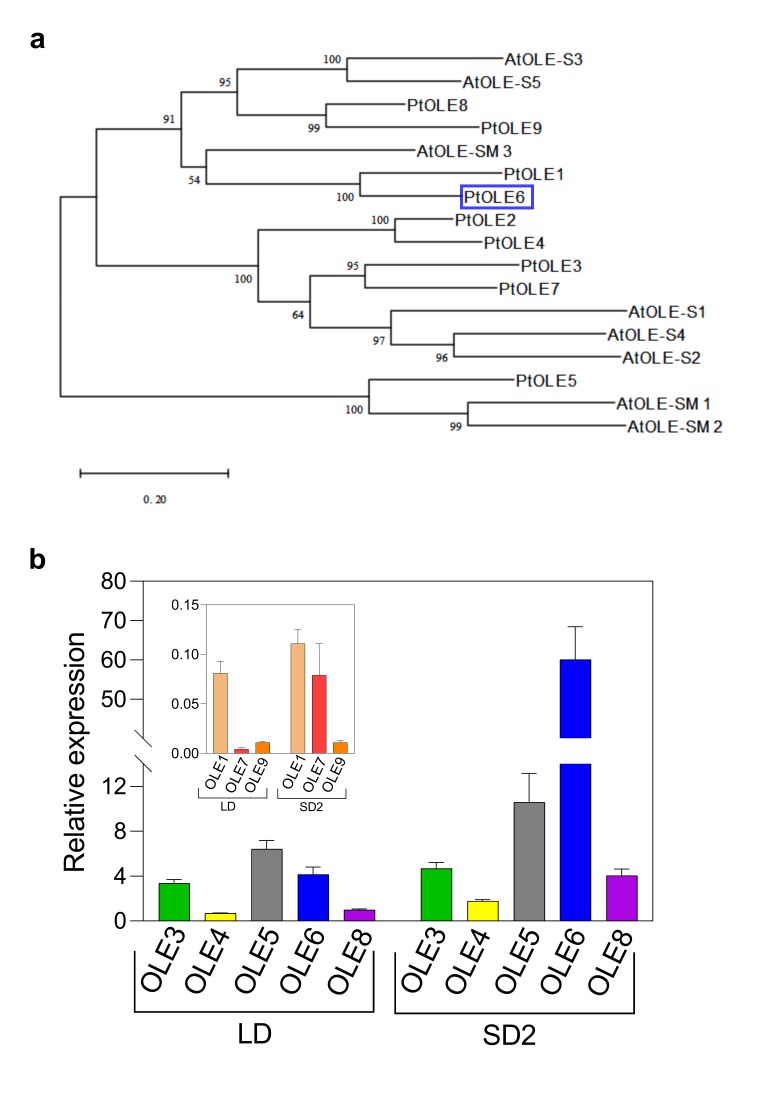
*OLEOSIN* gene identification and expression. (**a**) Phylogenetic analysis was performed with full-length OLEOSIN protein sequences of *Populus trichocarpa* (Table S1) and *Arabidopsis thaliana* (Table S3). Bootstrap percentages are shown on the dendrogram branch points. *Pt*OLE6 is boxed. (**b**) Relative expression of *Populus OLEOSIN* genes (*OLE1-9*) under long days (LD) and after two weeks in short days (SD2) coinciding with lipid body accumulation in developing terminal buds. Inset shows genes expressed at low levels. Fold changes are presented relative to *OLE8*, set to 1 in LD.

To investigate LB motility and PD targeting we used the LB marker-protein OLEOSIN. First, we used *Arabidopsis* OLEOSINs for genome-wide identification of homologues in *Populus trichocarpa* genome and carried out a phylogenetic analysis with the nine *Populus* OLEOSINs and eight *Arabidopsis* OLEOSINs and OLEOSIN-family proteins (Figure 2a). Our qPCR gene expression analysis confirmed that *OLEOSIN* genes are expressed in apices and developing buds. The data show that all identified *OLEOSIN* genes, except *OLE2*, were expressed in apices, although the expressions of *OLE1*, *OLE7,* and *OLE9* were very low (Figure 2b). When plants were exposed to a short photoperiod, which initiates terminal bud formation, most *OLEOSIN* genes were upregulated (Figure 2b) concomitant with the accumulation of LBs. In proliferating apices, expression was highest for *OLE5*, followed by *OLE6, OLE3, OLE8,* and *OLE4*, but in developing terminal buds, expression of *OLE6* was dramatically upregulated. As the expression of *OLE6* correlated best with the extent of LB-accumulation in buds, we selected *OLE6* for further experimentation.

### 2.3. Transgenic Expression of a Selected Populus OLEOSIN Gene

**Figure 3:**
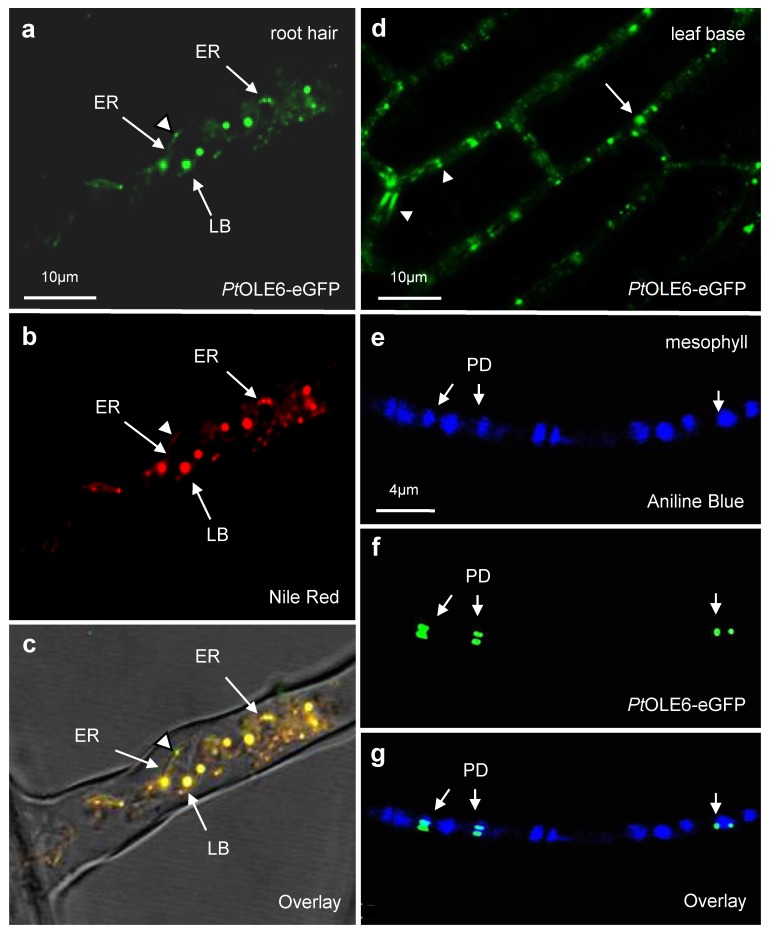
Transformation of *Arabidopsis* with *PtOLE6-eGFP.* (**a**–**c**) Root hairs, (**d**) leaf base, (**e**–**f**) mesophyll. (**a**) *Pt*OLE6-eGFP, and (**b**) Nile Red stained lipid bodies (LB) co-localize (**c**, overlay). LBs at different growth stages appear associated with a membrane system, putatively endoplasmic reticulum (ER). Arrowhead indicates presence of OLEOSIN protein, prior to lipid deposition. (**d**) *Pt*OLE6-eGFP localizes to plasmodesmata (PD) in cells at the leaf base. Arrowheads indicate punctate and sandwich pattern at PD. Arrow indicates LB. (**e**–**f**) Callose, stained with aniline-blue (**e**) and *Pt*OLE6-eGFP (**f**), co-localize to subsets of plasmodesmata (PD) (**g**, overlay). *Pt*OLE6-eGFP-tagged LBs target PD orifices in neighboring cells that share the PD-channel, thereby creating typical doublet spots.

We next aimed to investigate LB formation, localization, trafficking, and PD targeting. As the minute cells of the SAM do not lend themselves well to tracking LB movement, we instead used *Arabidopsis*. We genetically transformed *Col-0* plants with an *eGFP*-fusion of the short day-upregulated gene *PtOLE6* and selected two homozygous lines (OLE6-eGFP #1 and OLE6-eGFP #2) for further investigation. *Pt*OLE6-eGFP exclusively localized to LBs, and eGFP-tagged LBs were observed in all cells of the root and shoot of the transformed *Arabidopsis* plants (Figure 3). Root hairs were studied extensively, as they provide an unobstructed view of a single cell. In root hairs, *Pt*OLE6-eGFP was present as brightly fluorescent spherical bodies, somewhat variable in size, lining an apparent membrane system. In addition, numerous tiny and less brightly fluorescent spots were visible around it (Figure 3a). Staining with the neutral lipid stain Nile Red revealed an identical pattern (Figure 3b), and the overlay showed that the eGFP-tagged spheres co-localized with the Nile Red stained LBs (Figure 3c). The membrane system is likely to be ER, as it is associated with LBs at different stages of growth. If so, the tiny green fluorescent spots could possibly represent newly synthesized *Pt*OLE6-eGFP that had not yet diffused into a TAG nucleation site (Figure 3a–c).

### 2.4. PtOLE6-eGPF Tagged LBs Target Plasmodesmata

Previous data showed that LB-localized enzymes, a subclass of 1,3-β-glucanases referred to as γ-clade GH17 family proteins, targeted PD in *N. Benthamiana* leaves [52]. As root hairs are single cells that lack neighbors, except a few at the root hair base, we investigated leaf cells to assess the capacity of LBs to target PD, because leaf cells are interconnected by hundreds of PD. PD-targeting was variable between plants and cell types. In parenchyma cells at the leaf base, *Pt*OLE6-eGFP (OLE6-eGFP #1) was targeting numerous PD in all cell walls, resulting in walls speckled with fluorescent punctae and patches in a pattern that is typical for PD (Figure 3d). The patches show a strong resemblance to those originating from the transient expression of the LB-associated GH17-eGFP, which were sandwiching the PD [52]. In the larger mesophyll cell of Figure 3e–g, fewer PD were targeted, but fluorescent bodies precisely co-localized with the PD marker aniline blue (Figure 3e–g). Aniline blue fluorescence visualizes PD-associated callose, which is present in variable amounts in the cell wall surrounding the PD channel to regulate its size exclusion limit. Whereas aniline blue stained PD as a single blot, covering the PD channel and the surrounding cell wall collar, the fluorescence of *Pt*OLE6-eGFP was present in tiny but distinct punctae at the periphery of the PD orifices (Figure 3e–g), suggesting interaction with molecular recognition sites. Additionally, *Pt*OLE6-eGFP-fluorescence was present in small patches that sandwich both ends of PD in the adjoining walls of neighboring cells (Figure 3d–g), suggesting membrane association.

### 2.5. PtOLE6-eGPF Tagged LBs are Motile

We first monitored LB motility in root hairs, as they provide an unobstructed view. LBs were either free cytosolic bodies or seemingly attached to dynamic strands (Movie S1). In the main axis of the root many LBs showed vigorous directional movement, while others appeared to oscillate or showed Brownian movement (Movie S2). To assess if LB movement involved streaming ER or the cytoskeleton, we carried out pharmacological experiments with the microtubule depolymerizing drug oryzalin (10 µM) and the F-actin depolymerization drug cytochalasin D (cytoD, 40 µM). Oryzalin did not visually affect the movement of LBs, which was generally comparable to that in controls (Movie S3). In contrast, using a medium with cytoD arrested all directional LB trafficking, and only residual wobbling was visible (Movie S4). Washing the cytoD-treated seedlings in water restored LB movement (Figure S1). Together, these observations were a first confirmation that cytosolic free LB trafficking requires F-actin, and probably not microtubules.

### 2.6. Tracking LB Displacement and Velocity

**Figure 4:**
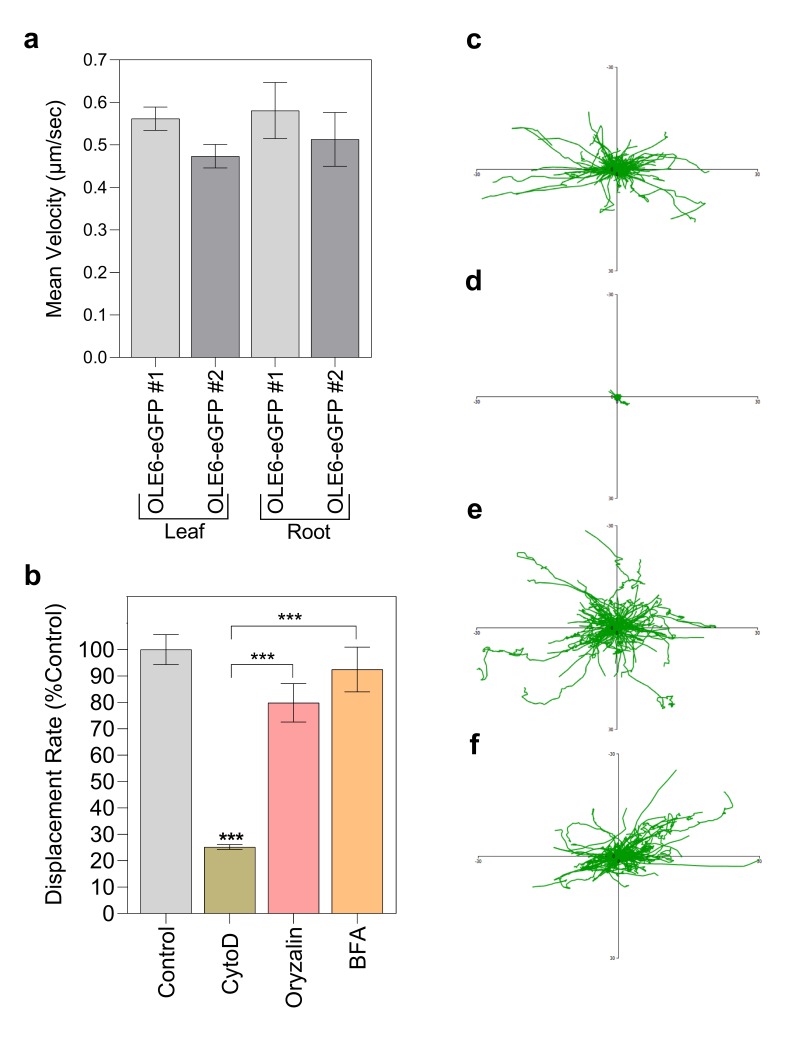
Movement of lipid bodies in *PtOLE6-eGFP-*overexpressing plants treated with different pharmacological agents. (**a**) Mean velocity of lipid bodies in leaf epidermal and root cells of two independent *PtOLE6-eGFP*-overexpressing lines OLE6-eGFP # 1 and OLE6-eGFp # 2 (± SE). (**b**) Mean displacement rates of the lipid bodies in leaf epidermal cells of OLE6-eGFP #1 treated with the indicated pharmacological agents and expressed as percentages of the control treatment (0.45 µm/s) (± SE). (**c**–**f**) Representative tracks of lipid bodies present in control (**c**), cytochalasin D (**d**), oryzalin (**e**), and brefeldin (**f**) treated plants in a time serie, obtained with the CLSM and plotted to a common origin. Asterisks indicate significant differences compared to control and between treatments (*** *p* < 0.001).

Although in cells of the main root some LBs were observed to target PD, the frequency of PD is very low in these cells because they become diluted during cell wall elongation. To investigate PD targeting, we therefore included leaf epidermal cells, which have numerous PD to neighboring cells. We tested the velocity and the displacement of LBs (Figure 4). To increase accuracy and avoid all perceptual bias, we used a Volocity software program (Velocity 6.5.1) that automatically tracked multiple LBs in the focal plane. The average velocity in both leaf epidermal cells and root cells in two independent *PtOLE6-eGFP-*overexpressing lines was around 0.5 µm s^−1^ (Figure 4a). Next, we performed pharmacological experiments in which we measured how displacement rates in epidermal cells of leaves were affected by the cytoskeletal drugs oryzalin, cytoD, and brefeldin A (BFA, 50 µM) (Figure 4b). Displacement rate was expressed as a percentage of the controls. Moreover, all fluorescent spots in the focal plane were tracked in a series of movies, and the data were depicted as an aggregate in which each particle had the same starting point.

Relative to the controls (Figure 4c; Movie S5), oryzalin and BFA had small inhibitory effects on mean displacement rates, but the effects were not statistically significant (Figure 4b; Movies S7 and S8), and the aggregate of LB tracks were very similar to that of the control (Figure 4c). As anticipated, the F-actin depolymerization drug cytoD had a very strong effect on the LB displacement rate, reducing it by around 75% (Figure 4b; Movie S6), while aborting virtually all LB tracks, except for some very short ones (Figure 4d). Increasing the concentration to 60 µM did not add to the effect (Figure S2).

Somewhat surprisingly, cytoD treatment did not reduce the displacement rate to zero, which could be due to high frequency wobbling, as their mean velocity was still considerable (Table S2). However, collectively, the results suggest that directional LB trafficking is on F-actin.

### 2.7. Tracking LB Displacement and Velocity in a 3KO Myosin (xi-k/1/2) Mutant

**Figure 5:**
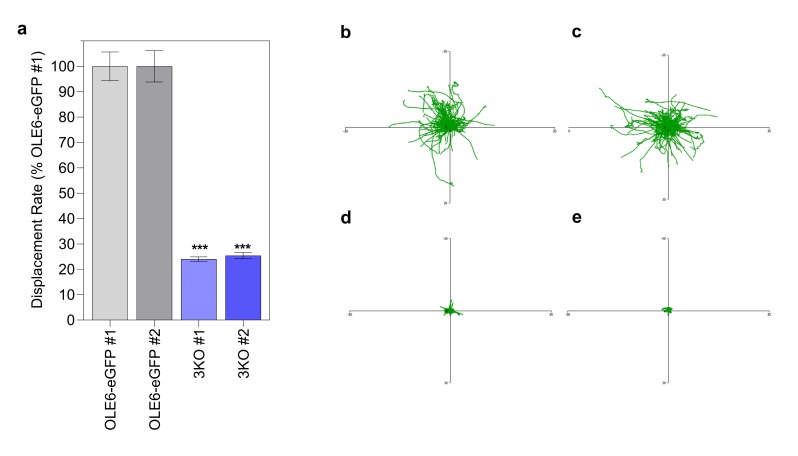
Movement of lipid bodies in *PtOLE6-eGFP*-overexpressing myosin 3KO plants. (**a**) Mean displacement rates of the lipid bodies in two independent *PtOLE6-eGFP* expressing lines in the myosin3ko background expressed as percentages (± SE) of those in the OLE6-eGFP #1 (0.45 µm/s). (**b**–**e**) Representative tracks of lipid bodies present in OLE6-eGFP #1 (**b**), OLE6-eGFP #2 (**c**), 3KO #1 (**d**), and 3KO #2 (**e**) in a time series obtained with the CLSM and plotted to a common origin. Asterisks indicate significant differences between control and the mutants (*** *p* < 0.001).

As trafficking of LBs on the actin cytoskeleton requires myosin motor proteins, we investigated whether movement was impaired in a triple myosin knock out mutant. Myosin XI is the major myosin involved in organelle trafficking in plants, with the three myosins XI-K/1/2 being the most important contributors [70]. We therefore overexpressed *PtOLE6-eGFP* in the triple KO mutant *xi-k/1/2* and selected homozygous lines for the investigations. The results showed that relative to controls (Figure 5b–c; Movies S5 and S9), for the two independent transgenic lines (3KO #1 and 3KO #2) that lacked myosins XI-K, XI-1, and XI-2, the displacement rate of LBs was reduced (Figure 5a) to levels almost identical to those in cytoD-treated leaf epidermal cells (Figure 4b). The aggregated LB tracks (Figure 5d–e) likewise showed that virtually all LB tracks were aborted, although some smaller local tracks were still present. As argued above, some of the remaining displacement is possibly due to high frequency residual wobbling or some unidentified mechanism, as the velocity was not completely abolished (Table S2; Movies S10 and S11).

## 3. Discussion

Investigations of LB biology have focused almost solely on seeds due to their economic importance [71]. In contrast, LBs in vegetative tissues have been inadequately studied, even though they might be important in plant development. Moreover, perennial buds contain considerable amounts of LBs [47]. This should not come as a surprise, as both seeds and buds establish dormancy and remain inactive until the conditions are beneficial for growth. In both seeds and perennial buds, LBs are loaded with neutral lipids that provide energy and lipid precursors for membrane biogenesis during germination and bud break. In addition, LBs in buds store enzymes that are crucial in dormancy breaking [52].

Although cells of mature buds are crowded with LBs, the SAM also possessed cytosolic LBs, albeit a low number (Figure 1d), as suggested before [51]. This is important, as LBs have mostly been identified from differentiated or determinate structures [71], whereas SAM cells are vigorously dividing. Like in animal systems [72], LBs in the SAM might have an antioxidant role, protecting the resident stem cells, in addition to a signaling function [5,52]. In the shoot apical meristem and the meristems of young developing buds, these LBs appeared as free, putatively mobile cytosolic bodies. Only occasionally were LBs observed in proximity to ER strands, perhaps tethered to them (Figure 1f). The solitary LBs were spherical, with a smooth monolayer and a homogeneous lipid matrix, while signs of coalescence were completely absent. In contrast, the LBs in mature buds appeared somewhat enlarged, and crowded the cytoplasmic space around the nuclei (Figure 1e). As buds, in contrast to orthodox seeds, do not completely desiccate during maturation [68], they might retain some metabolic activity. It seems possible, therefore, that the slight enlargement of LBs during bud development is based on the continuation of TAG biosynthesis at free cytosolic LBs [28]. In mature buds, some LBs might show coalescence (Figure 1e,g), which could be caused either by LB expansion and the resulting changes in LB surface tension [4,10], or by gradual degradation of OLEOSINs [73,74].

The most abundant LB protein in desiccation-tolerant seeds is OLEOSIN, and although OLEOSINs are also present in pollen grains, tapetum cells, and some leaf cells [71], in general they are considered seed-specific [38]. Nonetheless, the current data show that eight of the nine *OLEOSIN* genes that we identified in the *Populus* genome were expressed in the growing apical meristem and in young buds (Figure 2b). That the proliferating SAM expressed *OLEOSIN* genes at a low level is in retrospect not surprising, considering that they also produced some LBs. During early bud development, the expression of *OLEOSINs* was upregulated concomitantly with the accumulation of LBs. Among them, the gene *PtOLE6* was most strongly expressed. These data corroborate the understanding that many parallels exist between buds and seeds, although some differences are also apparent. In brief, while the LBs that pack mature buds (Figure 1e) might mostly function as stores of energy and lipid precursors for membrane biogenesis, the LBs in meristems (Figure 1d) and young developing buds are potentially mobile, functioning in transport, organellar interaction, and signaling. In non-seed tissues the scanty LBs are commonly regarded as detoxification refuges [71], and a function in transport and organellar interaction has remained underexplored. However, in meristems, and even in partially desiccated and fully dormant buds, LBs can be displaced toward PD. During dormancy release, LBs deliver the callose-hydrolyzing enzyme 1,3-β-glucanase to PD, which results in the removal of the callose-deposits in DSCs that were installed during dormancy establishment. This is important, as it re-opens the PD channels, thereby reestablishing the symplasmic communication network that is required for meristem function [5,47]. Yet, in proliferating meristems, LBs potentially contribute to the continuous exchange of signals and metabolites via PD that underlies primary morphogenesis [47,53,54,55,75].

The data provide evidence that *Pt*OLE6-eGFP-tagged LBs are mobile and able to target PD (Figure 3d–g; Movies S1,S2, S5 and S9). Considering such functional roles, it seems plausible that LBs in plant cells are enriched with hundreds of peripheral proteins, like those in mammalian cells [42,58,76]. In several animal systems, LBs traffic vigorously and directionally along microtubules to various intercellular destinations, motorized by kinesins and dyneins, and distributing lipids, lipophilic signals, and proteins [43,56,57,58,59]. In plant cells, organellar movement, including that of mitochondria and peroxisomes, has been shown to involve actin [66,67]. Yet, it remained unknown how LBs move in plant cells. The current data provide evidence that LBs are trafficked directionally by the actomyosin system, and not by microtubules as in animal cells. While at the base of some shorter root hairs, some of the LBs were immobile, possibly attached to the ER (Figure 3a–c), in most other cases the majority of the LBs moved vigorously along the length of root hairs, possibly following cytoplasmic strands (Movie S1). Pharmacological treatments excluded the possibility that LBs traffic on microtubules, as oryzalin did not affect it visually (Movie S3). In contrast, cytoD arrested LB trafficking (Movie S4), while washing restored it (Figure S1).

The automated recording and quantification of LB movement showed that the mean LB velocity in root and leaf epidermal cells was quite similar, around 0.5 µm s^−1^ (Figure 4a). Relative to the controls, the LB track aggregate in leaf epidermal cells was hardly influenced by treatment with oryzalin or BFA (Figure 4c,e,f), whereas cytoD virtually eliminated them (Figure 4d). Although the relative displacement rate showed that after cytoD treatment a residual LB movement of around 25% persisted, the LB track aggregate only displays a few very short LB tracks (Figure 4d). The residual movement therefore might represent the high-frequency wobbling that was observed in the CSLM (Movie S6).

Together, this clearly shows that F-actin is the major requirement for LB movement. Nonetheless, microtubules might interfere with the actin system, as oryzalin had a small effect on the LB displacement rate, although not a statistically significant one (Figure 4b). In pollen tubes, microtubules reduce the velocity of mitochondria trafficking [77], whereas the present data suggest that microtubules do not inhibit but rather mildly facilitate LB movement. It is therefore tempting to think that, like in mammalian systems, some microtubules might locally mediate nucleation and assembly of F-actin with the help of formins [78,79]. As in plant cells actin polymerization can also start from microtubules [80], this could facilitate actin-mediated LB transport, and explain the slightly reducing effect of oryzalin on LB trafficking. The recent finding that myosin XI-K co-localizes with microtubules in the forming cell division plane [81] suggests that there is interaction between the actin and microtubular cytoskeleton. Nonetheless, as the microtubule depolymerizing drug oryzalin did not clearly reduce LB trafficking in our experiments, we conclude that the actomyosin system is responsible for LB motility, while microtubules may have an indirect promoting effect.

Actin-based transport of LB requires myosins, which are processive motors [82]. Myosin XI heavy chains possess an actin-binding motor domain that hydrolyzes ATP, a neck region that functions as a lever, a coiled-coil domain, and a globular tail that can bind certain organelles [66]. *Arabidopsis* has 13 class XI myosins, which exhibit cell-specific expression patterns [67,83]. Of these, myosin XI-K is the most critical, and is involved in trafficking of mitochondria, peroxisomes, and endomembrane vesicles along F-actin, whereas XI-1 and XI-I contribute redundantly [65,84]. The current data demonstrate that class XI myosins are also responsible for the directional trafficking of LBs, as LB tracks were virtually abolished in the *Arabidopsis* 3KO *xi-k/1/2* mutant (Figure 5a,d,e). Previously, it was shown that in the 3KO mutant the processive transport of most Golgi stacks and peroxisomes was eliminated [70].

Recently, the interpretation of organellar movement has been subjected to a re-evaluation [65,84]. An often-adopted model depicts organelles as being trafficked by class XI myosins in combination with organelle-specific myosin receptors. In support of this model, class XI myosins can be associated with peroxisomes, mitochondria, and other cell organelles [85,86,87,88]. In an alternative model, there are only few *bona fide* myosin cargoes, whereas other organelles move passively. For example, myosin XI-K was shown to bind endomembrane vesicles via receptors of the myosin-binding protein (MyoB) family, transporting them on F-actin, and dragging other organelles passively in its wake [84,89]. We observed that LBs frequently moved in opposite directions in closely aligned paths, suggesting that they were not passively dragged by cytoplasmic streaming, but instead trafficked processively along F-actin cables. Notably, proteins with the MyoB1/2-specific myosin-binding DUF593 domain are hardly, or not at all, present in leaves and apices [89]. As shown for *Arabidopsis* [81], the function of myosin XI in the shoot apical meristem of *Populus* might also require different adaptor proteins. The observed antiparallel movements of LBs also exclude that the *Pt*OLE6-eGFP-tagged LBs moved on streaming ER [90], which could be driven by myosin XI [91]. We therefore suggest that LBs in *Arabidopsis* leaves are trafficked directionally and individually by the actomyosin system and propose that a similar mechanism is used in *Populus* meristems.

That *Pt*OLE6-eGFP-tagged LBs traffic directionally on F-actin to PD is not only supported by their localization at PD, but there is also circumstantial evidence that F-actin and myosin are involved in PD functioning. Firstly, both F-actin and myosin VIII have been immunolocalized to PD at pitfields [92,93,94], and actin actively regulates the size exclusion limit of PD [95]. Secondly, the actin-binding protein complex Arp2/3 complex, which nucleates actin filaments, localizes to PD [96,97,98]. Moreover, the actomyosin system is directly implicated in the movement of viruses toward and through PD [99,100]. For example, the Grapevine fanleaf virus produces movement protein-derived infection tubules through PD, mediated by the PD receptors PDLP (plasmodesmata located proteins), to allow the virus to move from cell to cell [101]. Blocking the function of myosin XI by selectively overexpressing its tail domain suppressed viral tubule formation and cell-to-cell movement. The implication is that the virus highjacks an existing myosin-dependent mechanism to traffic protein to PD. As shown for tobacco mosaic virus, replication complexes move on the ER-actin network to PD, driven by myosin XI-K/2, whereas final delivery to PD requires myosin XIII [100]. Movement from cell-to-cell may require severing of PD-actin, as in the PD channel actin provides scaffolding elements that restrict the channel size [102].

The present experiments show that LBs and their cargo can traffic on the actomyosin system to PD orifices. Although myosins XI-K/1/2 are the major myosins involved, other myosins may contribute because in the 3KO mutant some *Pt*OLE6-eGFP-tagged LBs can still be found at PD. It remains unclear whether OLEOSINs or some peripherally attached proteins are responsible for LB targeting to PD. It will be a challenge to establish what happens once a LB arrives at the PD gate, and the nature of the LB interaction with the PD orifice. As LBs can undergo hemi-fusion with phospholipid bilayers [43], we hypothesized earlier that LBs might interact with mobile membrane remorin-containing rafts at the cytoplasmic leaflet of the plasma membrane, and then diffuse laterally to the PD [5,52,53,75]. This possibility is supported by the observation that eGFP-tagged LBs can “collapse” and spread out as small patches that sandwich the PD channel (Figure 3d). Finally, it would be of great interest to establish to what degree the LB proteome overlaps with the PD proteome. Both LBs and PD are not unit structures, and their compositions are dynamically regulated in individual cells. It might turn out that LBs not only deliver lipid precursors for membrane biogenesis, but also components that become integrated into the fabric of the PD architecture, and signaling molecules that move to neighboring cells.

## 4. Materials and Methods

### 4.1. Plant Material

Hybrid aspen (*Populus tremula* × *Populus tremuloides*) clone T89 was micro-propagated in vitro for 5 weeks at 20 °C, planted in a mixture of soil/peat and perlite (4:1, *v*/*v*), fertilized with 4 gL^−1^ Osmocote, grown in a greenhouse at 20 °C and 60% relative humidity, and watered twice a day. Natural daylight was supplemented by mercury-halide lamps to a level of 200–250 µmol m^−2^s^−1^ at 400–750 nm (Osram) to obtain an 18 h long photoperiod. When the plants had reached a height of 80–100 cm, half of them were moved to a 10 h short photoperiod. This initiated the development of terminal buds, promoted LB accumulation in terminal buds, and further enhanced accumulation in axillary buds.

### 4.2. RNA Extraction and qPCR

Apices were collected from 9 plants, pooled into 3 biological replicates, and immediately frozen in liquid nitrogen. RNA was extracted from frozen samples, 0.2–0.3 g, and analyzed with quantitative RT-PCR as described before [103]. Gene-specific primer sequences for qPCR analysis were designed using Primer3 (http://frodo.wi.mit.edu/primer3). The list of primers and genes used for quantitative real time PCR (qRT-PCR) is presented in Table S1.

### 4.3. Generation of Arabidopsis Transgenic Lines

*PtOLE6* entry clone was constructed using Gateway™ technology (Invitrogen) and verified by sequencing (*OLE6* fwd 5’ CACCATGCCTGATCGATCAAGG; *OLE6 Rev* 5’ AGAAGTTTGAGT CTGACTAGAT). The binary vector expressing 35S promoter driven *PtOLE6-eGFP* fusion protein was constructed via the LR-reaction using the above-mentioned entry clone and destination vector pK7FWG2 [104] and later transformed into the *Agrobacterium tumefaciens* strain GV3101 (pMP90). *Arabidopsis thaliana*, ecotype Columbia (*Col-0*), and a myosin triple knockout (3KO: *xi-k/xi-1/xi-2*) line [70] were transformed by the floral dip method [105]. Transgenic lines were selected by segregation analysis, and two homozygous independent lines, each in the *Col-0* background (here referred to as OLE6-eGFP #1 and OLE6-eGFP #2) and 3KO background (here referred to as 3KO #1 and 3KO #2) were obtained.

### 4.4. Light and Electron Microscopy of LBs in Populus

Shoot apices and AXBs were fixed chemically overnight at 4 °C in 2% (*v*/*v*) glutaraldehyde and 3% (*v*/*v*) paraformaldehyde in 100 mM phosphate citrate buffer [47]. In brief, tissues were infiltrated gradually with LR white resin (LRW) of increasing concentration (30%–70%), and subsequently kept for 4 d in 100% LRW. Polymerization was carried out at 55 °C for 24 h. Samples were cut longitudinally into 1–3 μm thick sections and stained with filtered Sudan Black (IV) (1% *w*/*v* in 70% ethanol) under continuous stirring at 50 °C. Subsequently, sections were cleared with 70% ethanol for 2 min, and mounted in water for light microscopic observation (Leica DM6B) and imaged with a digital camera (DMC4500). Ultra-thin sections (70–80 nm) were cut from the LRW embedded samples and observed with a transmission electron microscope (FEI Morgagni 268) as described earlier [52]. In addition, LBs were isolated from axillary buds, as described elsewhere [106], stained with Nile Red (0.5% *w*/*v* in acetone and stained for 5 min) and photographed using the Leica TCS SP5 confocal scanning laser microscope (CSLM).

### 4.5. Co-Localization OLE6-eGFP and Plasmodesmal-Callose

Fresh leaf material was isolated from transgenic *OLE6-eGFP* expressing *Arabidopsis* plants (OLE6-eGFP #1). Leaf tissue was submerged for 30 min in 10 mM 2-deoxy-d-glucose (2-DDG; Sigma-Aldrich) to inhibit induction of wound callose [107], cut down, and further incubated in the dark for ~1 h in 0.1 M K_2_HPO_4_ buffer (pH 9.5) containing aniline blue (0.01%) and 2-DDG (5 mM). Co-localization of *Pt*OLE6-eGFP and aniline blue-stained plasmodesmal callose was examined and photographed with the CSLM.

### 4.6. LB-Motility and Pharmacological Treatments

Transgenic seedlings were surface sterilized and germinated on half strength Murashige and Skoog medium (MS) with kanamycin. For pharmacological treatments, seedlings were grown for six days and subsequently submerged in liquid MS medium with DMSO (control), cytoD (40 µM), oryzalin (10 µM), or BFA (50 µM) for 15 minutes. Immediately thereafter, LB movement was imaged with a Leica TCS SP5 CSLM (63× oil immersion lens). Time lapse images of eGFP emissions were obtained from leaf epidermal cells and root cells, including a total of 256 frames at 1.3 s intervals.

LB velocity and displacement rates were calculated using the Volocity 6.5.1 software (http://www.quorumtechnologies.com/volocity) according to the recommendation of the software manual. All the LBs with a diameter of 1 µm^2^ and above were analysed, and their two-dimensional movements were recorded for every movie clip. The mean track velocity (µm/s) and mean displacement rates (µm/s) were calculated using a minimum of 11 movie clips of individual seedlings (*n* ≥ 11). In total at least 2613 individual LBs were analysed for each experimental variant. The exact number of videos, total number of LBs, mean velocity, and mean displacement rates are referred to in Table S2.

### 4.7. Statistical Analyses and Bioinformatics

One-way ANOVA followed by Tukey’s multiple comparisons test were performed by using Graphpad Prism Version 8.0.0 (www.graphpad.com). In addition to previously reported *OLEOSIN* sequence homologues [108] we have identified one more *OLEOSIN* (*OLE9*) by protein BLAST searches in GenBank and the *P. trichocarpa* genome v3.0 [109] database (http://www.phytozome.net). The MEGA6 program (http://www.megasoftware.net/) was used for clustalW multiple sequence alignments and phylogenetic analysis, with Neighbour Joining method on 1000 bootstrap replications. The *Populus* and *Arabidopsis* proteins of the phylogenetic analysis are presented in Tables S1 and S3, respectively.

## Supplementary Materials

Supplementary materials can be found at https://www.mdpi.com/1422-0067/21/4/1422/s1.
