# Supplementary material for: Plant Lipid Bodies Traffic on Actin to Plasmodesmata Motorized by Myosin XIs
Source: Int J Mol Sci. 2020 Feb 20;21(4):1422. doi: 10.3390/ijms21041422 (PMC7073070; doi:10.3390/ijms21041422)
Supplement: Supplementary file 1 [file ijms-21-01422-s001.zip › Supplementary Figures and Tables.pdf]

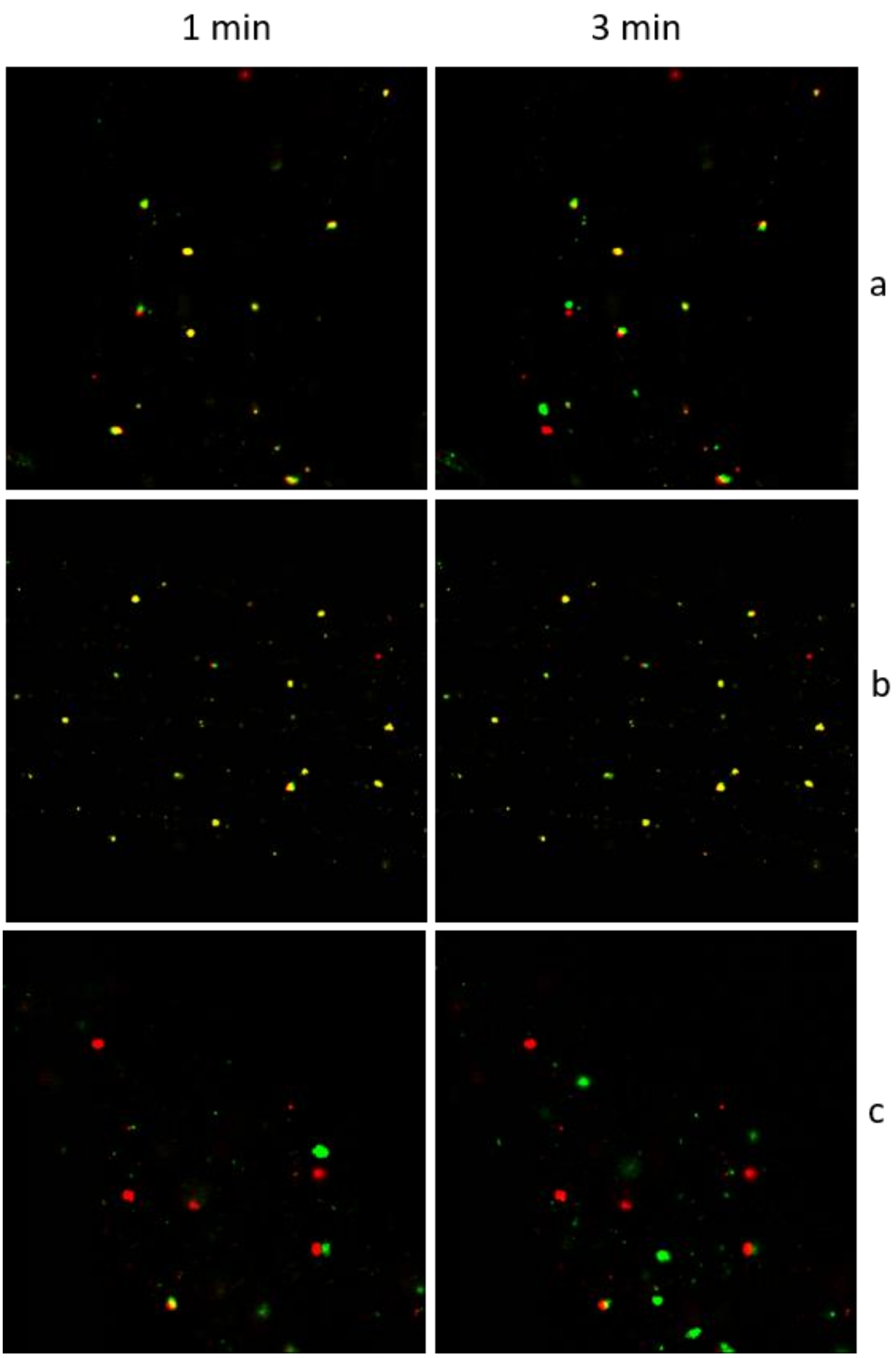

Figure S1. **Washing with water restored the cytoD mediated inhibition of LB motility.** Green/red overlay of two time lapse confocal images (1 and 3 minutes) of OLE6-eGFP #1 main root. Seedlings were treated with Control (a), 40µM cytD for 15 minutes (b) and washed with water after cytoD treatment (c). Note the near complete inhibition of LB movement (demonstrated by near-complete overlay of the two color signals) in cytoD treatment (b) and not in the water washed sample (c).

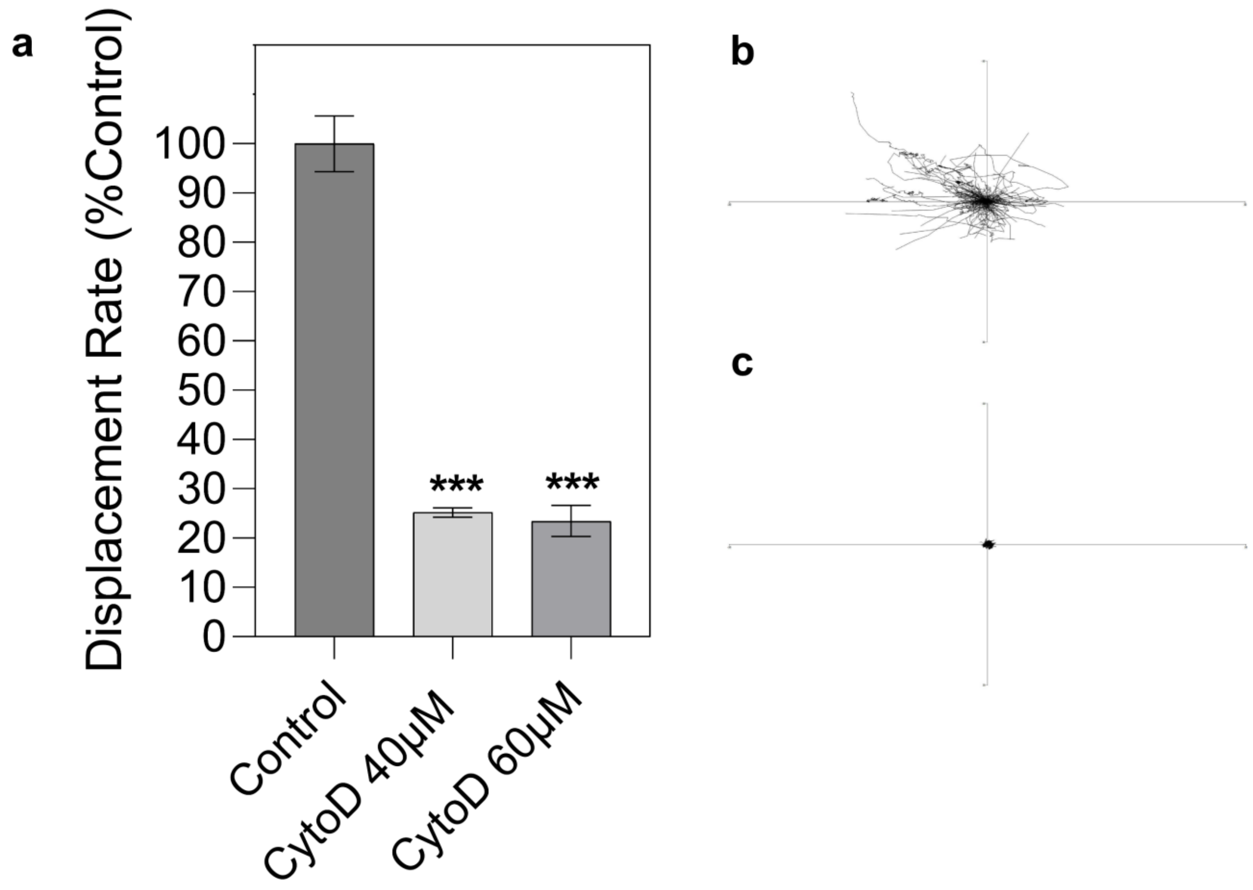

Figure S2. **Movement of lipid bodies in PtOLE6-eGFP overexpressing plants treated with different concentrations of CytoD.** (a) Mean displacement rates of the lipid bodies in leaf epidermal cells of PtOLE6-eGFP #1 treated with different concentrations of CytoD, expressed as percentage of those in the control treatment (0.45 µm/sec) (±SE). Representative tracks of lipid bodies present in the control (b), 60µM cytoD (c) in one time series obtained using the CLSM and plotted to a common origin. Asterisks indicate significant differences compared to control and between the treatments (***P<0.001).

Protein	Gene abb.	Locus name v3.0	Forward 5′-3′	Reverse 5′-3′
***OLEOSIN* genes**				
OLEOSIN1	*OLE1*	Potri.018G057800	GGTGCMGCACTGTTGGTGTT	GAATTGGTGTAGCCACGATCAA
OLEOSIN2	*OLE2*	Potri.015G082100	TGGATGGCCAGCTATATTCGA	GTGTCCTCCGTTTCGCTTGA
OLEOSIN3	*OLE3*	Potri.017G071800	CCTAGTAGGCACCCTGATCG	GAGGTCACTGCAAACCCAAT
OLEOSIN4	*OLE4*	Potri.012G083400	TTAGCATCCGGTGCTTTTGG	TGATCAAGCTCTGCGAATGG
OLEOSIN5	*OLE5*	Potri.012G059400	TCGGACCAGGTCGATTATGC	CATACTCCCTGGCGTAGTCCTT
OLEOSIN6	*OLE6*	Potri.006G234900	TTATGCTGGAGGGAGGATAGCA	TCTCACATTCTGACCGCACTCT
OLEOSIN7	*OLE7*	Potri.001G345800	GTCCCAGCTGCCCTTGTAATAG	AGGACCCTGTCAGTCCGAAA
OLEOSIN8	*OLE8*	Potri.001G080000	TTGCTGGTCATATTCAGTCCAGTT	AGGCCAAGAACCCCATAAGC
OLEOSIN9	*OLE9*	Potri.003G150600	ACCGGTGGGTCTCTTTTAGTTGT	ATGGAGTGGCTACGGTCAACA
**Housekeeping gene**				
ACTIN	*ACT*	Potri.001G309500	CGATGCCGAGGATATTCAAC	ACCAGTGTGTCTTGGTCTACCC

Table S1. ***P. trichocarpa* genes, identifiers and primer pairs used for qPCR analysis.**

S.No	Plant Material	Treatment	N[1]	N[2]	Mean Velocity ±SD (µm/sec)	Mean Displacement Rate ±SD (µm/sec)
1	OLE6-eGFP #1 Leaf	Control	12	2909	0.546 ± 0.106	0.456 ± 0.089
2	OLE6-eGFP #1 Leaf	CytoD 40µM	14	4327	0.201 ± 0.024	0,115 ± 0.016
3	OLE6-eGFP #1 Leaf	Oryzalin	11	5639	0.506 ± 0.133	0.364 ± 0.109
4	OLE6-eGFP #1 Leaf	BFA	16	6898	0.582 ± 0.227	0.464 ± 0.171
5	OLE6-eGFP #1 Root	Control	18	15638	0.580 ± 0.280	0.376 ± 0.181
6	OLE6-eGFP #2 Leaf	Control	14	5762	0.473 ± 0.103	0.366 ± 0.084
7	OLE6-eGFP #2 Root	Control	13	7973	0.512 ± 0.228	0.325 ± 0.177
8	3KO #1 Leaf	Control	23	6041	0.188 ± 0,027	0,109 ± 0.019
9	3KO #2 Leaf	Control	16	5260	0.197 ± 0.035	0,115 ± 0.022
10	OLE6-eGFP #1 Leaf	CytoD 60µM	12	2613	0.201 ± 0.069	0,117 ± 0.058

N[1]: Number of analysed videos
N[2]: Number of Analyzed Lipid body count (in N[1)]

OLE6-GFP #1: OLE6-eGFP overexpressor in *Columbia-0* background
OLE6-GFP #2: OLE6-eGFP overexpressor in *Columbia-0* background
3KO #1: OLE6-eGFP overexpressor in myosin 3 knockout background
3KO #2: OLE6-eGFP overexpressor in myosin 3 knockout background

Table S2. **Lipid body movement data.**

AT Gene IDs	Protein Name (Fang et al 2014)	Other names
At3g01570	OLE-S1	
At3g27660	OLE-S2	OLE4
At4g25140	OLE-S3	OLE1
At5g40420	OLE-S4	OLE2
At5g51210	OLE-S5	OLE3
At1g48990	OLE-SM1	
At3g18570	OLE-SM2	
At2g25890	OLE-SM3	

Table S3. ***A.thaliana*** **genes, identifiers used for the phylogenetic analysis.**
