# Supplementary material for: Plant Lipid Bodies Traffic on Actin to Plasmodesmata Motorized by Myosin XIs
Source: Int J Mol Sci. 2020 Feb 20;21(4):1422. doi: 10.3390/ijms21041422 (PMC7073070; doi:10.3390/ijms21041422)
Supplement: Supplementary file 1 [file ijms-21-01422-s001.zip › Supplementary Movies/Supplementary Movie legends.pdf]

Supplementary movie Legends:

Movie S1. Time-lapse confocal scanning microscopy of the *Arabidopsis* root hair stably expressing PtOLE6-eGFP in *Columbia-0* plants (OLE6-eGFP #1). The movie is presented at a rate of 10 frames per second.

Movie S2. Time-lapse confocal scanning microscopy of the *Arabidopsis* main root hair stably expressing PtOLE6-eGFP in *Columbia-0* plants (OLE6-eGFP #1). The movie is presented at a rate of 10 frames per second.

Movie S3. Time-lapse confocal scanning microscopy of the *Arabidopsis* main root hair stably expressing PtOLE6-eGFP in *Columbia-0* plants (OLE6-eGFP #1) treated with 10µM Oryzalin. The movie is presented at a rate of 10 frames per second.

Movie S4. Time-lapse confocal scanning microscopy of the *Arabidopsis* main root hair stably expressing PtOLE6-eGFP in *Columbia-0* plants (OLE6-eGFP #1) treated with 40µM CytoD. The movie is presented at a rate of 10 frames per second.

Movie S5. Time-lapse confocal scanning microscopy of the *Arabidopsis* leaf epidermis stably expressing PtOLE6-eGFP in *Columbia-0* plants (OLE6-eGFP #1). The movie is presented at a rate of 50 frames per second.

Movie S6. Time-lapse confocal scanning microscopy of the *Arabidopsis* leaf epidermis stably expressing PtOLE6-eGFP in *Columbia-0* plants (OLE6-eGFP #1) treated with 40µM CytoD. The movie is presented at a rate of 50 frames per second.

Movie S7. Time-lapse confocal scanning microscopy of the *Arabidopsis* leaf epidermis stably expressing PtOLE6-eGFP in *Columbia-0* plants (OLE6-eGFP #1) treated with 10µM Oryzalin. The movie is presented at a rate of 50 frames per second.

Movie S8. Time-lapse confocal scanning microscopy of the *Arabidopsis* leaf epidermis stably expressing PtOLE6-eGFP in *Columbia-0* plants (OLE6-eGFP #1) treated with 50µM BFA. The movie is presented at a rate of 50 frames per second.

Movie S9. Time-lapse confocal scanning microscopy of the *Arabidopsis* leaf epidermis stably expressing PtOLE6-eGFP in *Columbia-0* plants (OLE6-eGFP #2). The movie is presented at a rate of 50 frames per second.

Movie S10. Time-lapse confocal scanning microscopy of the *Arabidopsis* leaf epidermis stably expressing PtOLE6-eGFP in myosin 3KO plants (3KO #1). The movie is presented at a rate of 50 frames per second.

Movie S11. Time-lapse confocal scanning microscopy of the *Arabidopsis* leaf epidermis stably expressing PtOLE6-eGFP in myosin 3KO plants (3KO #2). The movie is presented at a rate of 50 frames per second.
